# Glyceraldehyde-3-phosphate Dehydrogenase Is a Multifaceted Therapeutic Target

**DOI:** 10.3390/pharmaceutics12050416

**Published:** 2020-05-02

**Authors:** Vladimir F. Lazarev, Irina V. Guzhova, Boris A. Margulis

**Affiliations:** Institute of Cytology of the Russian Academy of Sciences, 194064 St. Petersburg, Russia; irina.guzh@gmail.com (I.V.G.); margulis@incras.ru (B.A.M.)

**Keywords:** GAPDH, drugs, neurodegenerative diseases, cancer, diabetics, treatment

## Abstract

Glyceraldehyde-3-phosphate dehydrogenase (GAPDH) is a glycolytic enzyme whose role in cell metabolism and homeostasis is well defined, while its function in pathologic processes needs further elucidation. Depending on the cell context, GAPDH may bind a number of physiologically important proteins, control their function and correspondingly affect the cell’s fate. These interprotein interactions and post-translational modifications of GAPDH mediate its cytotoxic or cytoprotective functions in the manner of a Janus-like molecule. In this review, we discuss the functional features of the enzyme in cellular physiology and its possible involvement in human pathologies. In the last part of the article, we describe drugs that can be employed to modulate this enzyme’s function in some pathologic states.

## 1. Introduction

Glyceraldehyde-3-phosphate dehydrogenase (GAPDH) is one of the major housekeeping proteins, comprising approximately 2,000,000 molecules per cell and occurring in molar concentrations of about 0.4 μM [1]. With such a high content, the enzyme can reach its well-known functional diversity by interacting with miscellaneous protein partners as well as with DNA and RNA species [2].

GAPDH concurrently catalyzes the phosphorylation and oxidation of glyceraldehyde-3-phosphate to generate 1,3-biphosphoglycerate using NAD+ as the electron acceptor, resulting in the production of NADH. GAPDH is a homo-tetramer and one of the cellular proteins abnormally enriched by reactive sulfhydryl groups; this explains the unusually high aggregation capacity of the *S*-nitrosylated or oxidized protein. Importantly, these modifications have a significant impact on a great variety of neurodegenerative processes [3,4]. The enzyme catalyzes the glycolytic reaction resulting in the creation of macroergic products and NADH, which are used further in reactions of oxidative phosphorylation [5]. In addition, the activities of GAPDH may be regulated by redox reactions, for example *S*-thiolation, which appears to serve an adaptive function during exposure to an oxidative stress [6]. GAPDH is capable of functioning in the cell both in the enzymatically active, tetrameric form necessary for glycolysis, and in the dimeric or monomeric forms [7,8]. Moreover, the cellular localization of GAPDH is not limited to the cytoplasm, the protein is found in the nucleus and other intracellular organelles [9], including plasma membrane [10]. Multiple modifications of GAPDH, phosphorylation, oxidation and others, also contribute to the plethora of the enzyme’s activities in following processes of cellular physiology, including intracellular transport [11], cytoskeleton plasticity [12], transcription [13], heme chaperoning [14], protection of telomeric DNA [15], lactoferrin receptor activity [16] and some other functions. Additionally, a distinct, sperm-specific form of GAPDH is isolated, the main function of which is glycolysis, and impaired functioning may cause male infertility [17]. One of the features for which GAPDH is known around the world is its use as a loading control in hundreds of studies dedicated to the analysis of omics. It is of note, however, that this application is not always justified because the quantity of this enzyme has been shown to vary under stressful and other conditions [18,19]. The unfortunate discrepancy between the vital, multifunctional, and, at the same time, not so highly appreciated role of GAPDH in cell physiology has been marked by a few authors [2,20,21] and has prompted us to offer more facts proving the enzyme to be an important drug target.

Multiple activities of GAPDH in a eukaryotic cell can be linked to pro-survival or pro-apoptotic functions, depending on the enzyme’s state or protein environment, and in this review, we discuss both these activities and their relation to the physiology of normal and cancer cells. In the final part, we overview recent achievements in the development of drugs able to modulate the functions of the enzyme in human pathologies.

## 2. Glyceraldehyde-3-Phosphate Dehydrogenase (GAPDH) Functioning in Pathological States

GAPDH performs a huge number of functions in a cell and participates in many vital chemical cascades as a typical moonlighting protein. In addition to performing the functions that are necessary for normal cell physiology, GAPDH is involved in the cell’s response to various types of cytotoxic or damaging factors, such as oxidative stress [22,23], starvation [24], proteotoxic stress [25], toxicity of chemical agents [26], and others. These factors affect the conformation of GAPDH or even destruct its native tetrameric state and cause post-translational modifications of the enzyme. Such chemical modifications of GAPDH may strongly influence the activity of certain organelles, such as mitochondria, the transport system associated with the cytoskeleton, the proteostasis mechanisms related to autophagy, and some others. Although there is no data about a pathology wholly associated with the enzyme’s damage or deficiency, many neurological diseases are reported to partially implicate impaired, aggregating GAPDH or tumors, strongly depending on the energy supply promoted by this enzyme through the Warburg effect [27].

In order to form a clearer picture of the participation of GAPDH in pathological states, we divided the pathologies associated with the enzyme function into several groups (Table 1): (I) pathologies associated with pro-apoptotic GAPDH activity; (II) pathologies associated with impaired function of GAPDH; (III) pro-survival activity of GAPDH in cancer cells.

### 2.1. Pathologies Associated with Pro-Apoptotic GAPDH Functioning

GAPDH is well established as one of the molecules promoting apoptotic signaling in the cell nucleus. GAPDH may induce apoptosis using several distinct mechanisms. One of them is due to NO-induced S-nitrosylation of C152 residue on the GAPDH molecule (SNO-GAPDH), causing its interaction with E3 ubiquitin ligase Siah1, with the stabilization of the latter, and the intranuclear translocation of the complex [48]. Siah1 is involved in proteasome degradation of a number of nuclear proteins, in particular Ncor, which may presumably affect apoptosis [49]. Additionally, Siah1 causes p53-dependent apoptosis in K-562 and U-937 cells by using a TCP-1 chaperonin-based mechanism, proving that GAPDH may engage the versatile tumor suppressor distantly to initiate cancer cell death [50]. The suggestion that Siah1, translocated into the nucleus by GAPDH, is able to exert its ubiquitin ligase and pro-apoptotic activity was proved by the data indicating a clear correlation between the appearance of the GAPDH–Siah1 complex in the nucleus and the degree of cell death [51,52]. On the other hand, GAPDH may form a direct complex with p53, leading to the activation of AMPA receptor and significantly increasing both the expression of p53 and its phosphorylation at Ser46 [53]; this modification is meaningful because it was found to activate apoptosis [54]. Importantly, according to several reports intranuclear translocation of GAPDH correlates with p53 activation and transcription of BAX, PUMA and p21, leading to p53-dependent cell death [55,56]. Despite these convincing reports, a pathway of apoptosis that is predominantly affected by SNO-GAPDH-Siah1 nuclear translocation needs further elucidation. It was also shown that the nuclear translocation of GAPDH is mediated by acetylation of K117, K227 and K251 catalyzed by acetyltransferase P300/CBP-associated factor [57]. Another possible apoptosis-inducing mechanism is triggered by the interaction of GAPDH with VDAC1, which is located at the membrane of the mitochondria. This leads to the opening of permeability transition pores (PTPs) and to the depolarization of the mitochondria, as well as to the release of cytochrome C and of an apoptosis-inducing factor [58]. According to the recent data, the PTP opening may be caused by the denatured GAPDH as a result of S-nitrosylation of C152 residue; the replacement of C152 with alanine resulted in the blockage of cell death in response to nitrosative stress [59], which proves the importance of GAPDH nitrosylation in regard to the advancement of apoptosis. Of note, nitrosylation of GAPDH can also augment tau acetylation in the presence of Abeta1-42, which causes a violation in the microtubule association process and the amount of nitrosylated GAPDH is increased in post-mortem Alzheimer’s disease (AD) brains [60].

The ability of nitrosylated GAPDH to induce apoptosis also plays a significant role in diabetic retinopathy. Hyperglycemia, which remains an important factor in post-diabetes retinopathy, leads to a significant increase of nitrosylated GAPDH in retinal cells, which causes the intranuclear translocation of the enzyme and induction of apoptosis [33,61]. In addition, a high glucose level leads to an increase in the amount of Siah1, the formation of the GAPDH-Siah1 complex, and its nuclear translocation that causes apoptosis [62].

Another cause of GAPDH-mediated cell death is protein aggregation as a result of oxidative stress and the accumulation of reactive oxygen species [63]. The most probable site for the oxidation is the C152 residue located in the catalytic region of the enzyme molecule. Oxidation of C152 causes an inactivation of GAPDH and the formation of intra- or inter-molecular disulfide bonds [64,65]. Another mechanism triggered by the oxidation of M46 on the GAPDH molecule, which also leads to enzyme denaturation and the formation of aggregates through disulfide bridges [66]. Despite the repetitively demonstrated correlation between the formation of GAPDH aggregates and cell death, the mechanism of apoptosis induced by such complexes has not been studied in detail. On the other hand, there is a growing dataset indicating the initiation of apoptotic cascades during the formation of co-aggregates of GAPDH with mutant proteins including huntingtin [67,68], superoxide dismutase [69], beta-amyloid [70,71], alpha-synuclein [5,29], neuronal tau [72] and others. Apoptosis can also be caused by extracellular GAPDH aggregates formed due to massive cell death typical of traumatic brain injury. Recently, we showed a significant toxic effect of exogenous GAPDH aggregates on surrounding cells [28,73]. In this way, the formation of GAPDH aggregates facilitates further misfolding of the GAPDH, leading to the impairment of the enzyme molecule structure and ultimately a conversion to high molecular weight aggregates, which are usually cytotoxic and may trigger apoptosis [4,74].

Together, the data on GAPDH-mediated cell death indicate that the chemical modifications and translocation of the enzyme to the nucleus cause cytotoxic cascades. Consistently, it is believed that inactivated, aggregation-prone GAPDH can enhance such pathologies as Alzheimer’s disease [75], Huntington’s disease [76,77], Parkinson’s disease [78], diabetes [79], secondary damage after traumatic brain injury [28] and many others. Therefore, targeting specific sites on the GAPDH molecule may be useful for increasing the viability of cells subjected to proteotoxic factors, such as misfolded proteins in neurological diseases.

### 2.2. Pathologies Associated with Impaired Function of GAPDH

The key function of GAPDH is attributed to glycolysis, which means that the active tetrameric molecule catalyzes the conversion of glyceraldehyde-3-phosphate to 1,3-bisphospho-d-glycerate [80]. Chemical modifications and denaturation of GAPDH can slow down the glycolytic cycle and disrupt the cellular metabolism [81]. Therefore, it is not surprising that a violation of glycolysis can aggravate a number of pathological conditions. An impairment of glycolysis may be caused by direct binding of the enzyme by other biomolecules, e.g., by blocking its glycolytic activity. For instance, this happens during the development of dentatorubral–pallidoluysian atrophy (DRPLA); DRPLA protein binds the GAPDH and inhibits glycolysis [36,68]. Another reason for a GAPDH-mediated violation of glycolysis is an autoimmune reaction that brings about the appearance of anti-GAPDH antibodies. This phenomenon was shown for the sperm-specific form of GAPDH that is necessary for sperm motility [38]. An analysis of antibodies to the sperm-specific form of GAPDH showed their increased titer in serum in the group of patients with immune infertility in comparison with that in the fertile group. The destruction of the barrier that provides testes immune privilege can lead to penetration of autoimmune anti-GAPDH antibodies into the testes tissue and cause male infertility [37,82]. Moreover, the oxidation of the GAPDH sperm-specific form caused by high glucose levels leads to reduced sperm motility [83]. In support of the importance of active GAPDH for male fertility, it is worthy of note that a recent publication demonstrated the correlation between the expression level of sperm-specific GAPDH form and sperm quality. The low level of GAPDH expression correlates with the decreased level of sperm motility and enhanced asthenozoospermia in patients [17].

Another pathology associated with a substantial deficit of glycolysis is age-related cataract; at least partially, the deficit is related to the reduced level of GAPDH in lens fiber cells [84]. This reduction may cause an impairment of the ATP accumulation in cells. ATP is necessary for a wide spectrum of physiological processes, including the correct function of the chaperone system. Reducing ATP content may lead to a deterioration in the chaperoning of alpha-crystallin and its incorrect folding, resulting in acceleration of retinal clouding [41]. Finally, the data obtained on human aortic smooth muscle cells shows that the down-regulation of GAPDH glycolytic activity and subsequent ATP depletion caused alterations in arterial wall energy homeostasis leading to atherosclerotic pathogenesis [85].

The disturbance of energy metabolism due to inactivation of GAPDH is believed to contribute to the development of the selective damage of proximal tubular cells after ischemic renal injury. Poly(ADP-ribose) polymerase-1 inhibits GAPDH activity in hypoxia conditions through ADP-ribosylation of C152 on GAPDH molecule. Impairment in ATP metabolism caused by the inhibition of glycolysis, leading to the necrosis of LLCPK1 cells [42].

The implication of GAPDH in pathologies stemming from mitochondrial dysfunction has been demonstrated in experiments on models of ischemia, traumatic brain injury, and oxidative stress. GAPDH acts as a trigger for mitophagy in cells after ischemia. The enzyme associated with the mitochondrial membrane is necessary for the penetration of damaged mitochondria and their utilization in lysosomes. Phosphorylation of GAPDH at threonine 246 by PKCδ prevented GAPDH-mediated mitophagy, leading to the accumulation of damaged mitochondria in the cytosol, the release of cytochrome C, and apoptosis [86]. The binding of GAPDH to long polyglutamine tracts in Huntington’s disease also causes the disruption of mitophagy and probably contributes to the pathogenesis of Huntington’s disease. [43].

Recent studies have demonstrated the role of GAPDH in maintaining heme homeostasis; the enzyme was shown to be obligatory for inducible NO synthase (iNOS)-mediated heme insertion, since a mutant GAPDH (K227A) molecule almost completely inhibited the heme insertion [87]. NO-induced *S*-nitrosylation of GAPDH by C152 makes the enzyme unable to provide heme insertion in iNOS; interestingly, the C152A mutation in the GAPDH molecule protects iNOS heme insertion against the NO inhibition [87,88]. On the other hand, the binding of the heme by GAPDH, reported by the same group [14], significantly reduced the catalytic activity of the heme and, accordingly, its cytotoxicity [89]. Recently, histidine in the 53-th position was shown to be critical for the interaction between GAPDH and heme, since the H53A mutant had no binding activity [90]. The authors suggested that GAPDH together with Hsp90 chaperone, chaperones the unstable heme, thus increasing its bioavailability for inserting in proteins.

The implication of GAPDH in the regulation of inflammation is proved by data indicating that it participates in the maturation of cell catalase through the chaperoning of heme. Catalase is a heme-containing enzyme which is necessary for the processing of peroxide forms of oxygen. Impairment of GAPDH function led to the inhibition of catalase activity in the smooth muscle cells. It is suggested that the impairment of heme chaperoning caused by GAPDH nitrosylation contributes to the decrease of catalase activity which is typical of inflammatory processes, such as asthma [91].

Another possible way for GAPDH to affect the inflammatory process is through regulation of tumor necrosis factor (TNF) synthesis. GAPDH can bind TNF mRNA, which leads to post-transcriptional repression of TNF and generation of the anti-inflammatory effect [92]. Of note, the authors observed a similar interaction in THP-1 monocytes with a low level of glycolysis, and the intensification of glycolysis led to the destruction of the GAPDH–TNF mRNA complex and the activation of the inflammatory process. Such GAPDH-mediated pro-inflammatory cascades can occur after severe injuries and sepsis [44,93]. In these cases, blocking inflammation is a mandatory stage of therapy, and GAPDH is a potential drug target.

### 2.3. Pro-Survival Function of GAPDH in Cancer Cells

The mechanism of the anaerobic conversion of glucose plays a significant role in tissue regeneration and, certainly, in tumor growth [94,95]. Constantly persisting in conditions of metabolic stress, hypoxia, and starvation, cancer cells need to increase the capacity of their glycolysis mechanism by using the Warburg effect, e.g., by increasing the activity of enzymes involved in the function [96]. Herein, the rate of total glycolysis flux is determined precisely at the stage of conversion of glyceraldehyde-3-phosphate to bis-phosphoglycerate and is regulated by the active GAPDH tetramer [97]. Since GAPDH is necessary for the limiting step in glycolysis, the enzyme molecule plays a crucial role in the whole energy metabolism of a cancer cell. This means that a high level of GAPDH activity leads to elevated glycolysis intensity, enhancement of tumor growth, and, accordingly, a poor prognosis for patients [98]. While targeting glycolysis in cancer cells is a promising approach, there are limitations to such a therapy, related to the necessity for biomarkers to determine the conditions in which abrogating glycolysis would be effective. Recent studies showed that the intensity of glycolysis in tumor cells itself may be a marker for predicting the effectiveness of anticancer therapy based on the inhibition of GAPDH activity; moreover, in the case of high glycolysis levels, a therapy with a GAPDH inhibitor, koningic acid, was more effective than in the case of low intensity of glycolysis [99].

Hypoxia, which is one of major phenomena accompanying tumor growth, leads to an elevation of GAPDH expression through the activated Hif-1α transcription factor [100,101]. Additionally, a number of reports offer an obvious link between a high level of active GAPDH tetramer, the elevated mobility of cancer cells, and the markers of the epithelial-mesenchymal transition. Using the chromatin immunoprecipitation assay on colon cancer cells, it was proved that GAPDH directly interacts with the SP1 transcription factor, which leads to an increased expression of zinc finger protein SNAI1 (Snail), the master regulator of the epithelial–mesenchymal transition [21,46,102]. It is likely that the activation of GAPDH synthesis can be considered as a protective mechanism employed by tumor cells in conditions of hypoxia to regulate their metabolism and increase their viability. Of note, elevated expression of GAPDH leads to NF-κB-dependent activation of Hif-1, which causes an enhancement of vascularization and aggressiveness in non-Hodgkin’s B lymphoma cells [45].

Starvation entails the need to modulate the energy metabolism, and GAPDH is also implied in cell response to starvation. Starvation causes the translocation of GAPDH from the cytosol to other cell compartments, including the Golgi apparatus. Additionally, GAPDH was found to bind the ADP-ribosylation factor GTPase-activating protein 1, which leads to the inhibition of the COPI protein and to the suppression of transport from the Golgi compartment. Thus, under starvation conditions, GAPDH optimizes energy homeostasis and increases cell survival [103]. Another important mechanism triggered by GAPDH in starving tumor cells is autophagy linked to Sirt1 activation. Under conditions of glucose deficiency, AMP-activated protein kinase phosphorylates GAPDH at S122, causing the enzyme to translocate to the nucleus and bind to Sirt1. The interaction of the two proteins leads to activation of the Sirt1 which deacetylates Atg5 and Atg12 proteins, the principal components of the macroautophagy mechanism. Consequently, deacetylation activates both Atg, leads to initiation of the autophagy complex and, on the other hand, to the protection of HeLa carcinoma cells from caspase-independent cell death [104,105]. Importantly, GAPDH-mediated up-regulation of Atg5-Atg12 expression enhances cytoprotective autophagy, thereby antagonizing apoptosis [106]. In parallel, GAPDH stabilizes the activated Act1 kinase, leading to Bcl-xL overexpression and enhanced resistance to caspase-independent cell death [107].

In contrast to modifications that cause GAPDH nuclear translocation and lead to apoptosis, the phosphorylation of the enzyme (T237) by protein kinase Bβ was reported, which inhibits both the GAPDH transport and the apoptosis-promoting activity in OVCAR-3 ovarian cancer cells [47].

It is also worth mentioning that GAPDH was found to protect telomeric DNA in cancer cells subjected to chemotherapy. In particular, it was demonstrated that GAPDH molecules physically interact with telomeric DNA and protect it against rapid degradation in A549 cells treated with doxorubicin [15]. Moreover, GAPDH over-expression accelerated the aging process in breast cancer cells due to telomerase inhibition caused by an interaction between the GAPDH and the telomerase RNA [35]. The authors suggested that the inhibition of telomerase with an increase in the amount of GAPDH can lead to senescence. Opposing data were obtained on non-small cell lung cancer cells by Phadke et al., who showed that GAPDH depletion impaired glycolysis and led to cell cycle arrest through 5’-AMP-activated protein kinasemediated pathway [108]. Since the regulation of the senescence mechanisms by GAPDH is strongly dependent on the origin of tumor, this variability should be taken into account when choosing a therapeutic strategy.

## 3. Overview of GAPDH-Targeting Drugs

The numerous pro-apoptotic or, more generally, pro-pathologic effects of GAPDH have prompted researchers to seek substances blocking the neurotoxic activity of the enzyme. The first compound found to inhibit GADPH expression was the anti-dementia drug tetrahydroaminoacridine, or tacrine [109]. A few years later, the same group offered a new substance, (*S*)-1-[*N*-(4-chlorobenzyl) succinamoyl] pyrrolidine-2-carbaldehyde (ONO-1603), that also inhibited the overexpression of GAPDH mRNA and effectively delayed age-induced apoptosis in cultured neurons [110]. These substances, tacrine and donezepil (commercially available as Aricept), both known as cholinesterase inhibitors, demonstrated protective activity when applied to primary cultures of rat cerebral cortical and cerebellar granule cells [111] (see Table 2).

Another group of GAPDH-targeting substances includes an inhibitor of monoamine oxidase type B (MAO) –(-)deprenyl and its chemical derivatives (–)-desmethyldeprenyl, and a tricyclic deprenyl analog, CGP3466; these substances belong to the broad group of propargylamines. Selegiline, a trade mark of –(-)deprenyl, is a well-established drug for patients with Parkinson disease and is more widely applied in the treatment of pathologies associated with defects in the functioning of of dopaminergic neurons (see Table 2). Propargylamines were found to reduce apoptosis independently of MAO inhibition and exhibit a variety of neuroprotective effects; these activities are presumably due to the ability of propargylamines to bind GAPDH [112,113]. Molecular studies show that propargylamines exhibit anti-apoptotic effects in vitro and in vivo by keeping GAPDH in dimeric form and preventing its intranuclear transport and apoptosis [114]. This view is well in accord with earlier data indicating that GAPDH damage and dissociation on monomers occurs due to an increase in the hydrogen peroxide level, a by-product of an enzymatic reaction catalyzed by MAO [115]. Besides the link to GAPDH, it has been established that deprenyl and its chemical analogs activate superoxide dismutase and catalase in brain dopaminergic regions or in extra-brain tissues such as heart and kidneys [116]. More recently –(-)deprenyl was demonstrated in vitro to reduce the aggregation capacity of a GAPDH complex with mutant huntingtin [32] and to augment cell survival in models of polyglutamine expansion-related diseases [67].

Herein, we presented data evidencing that GAPDH, upon being oxidized or S-nitrosylated, can form aggregates alone or in complex with numerous mutant polypeptides; these complexes and/or co-aggregates are often toxic and therefore appropriate efforts are being made to inhibit the aggregation capacity of the modified enzyme. One such inhibitor was offered by Itakura and coauthors and constitutes a GAPDH aggregation inhibitor (GAI) decapeptide (Table 2). The GAI reduced GAPDH aggregation in a concentration-dependent manner, but did not affect GAPDH’s glycolytic activity or cell viability. Importantly, the peptide exhibited a protective effect against oxidative stress-induced cell death in human neuroblastoma cells, and so the authors suggested that GAI may become a safe drug for the treatment of neurodegenerative pathologies [117]. We started searching for anti-GAPDH aggregation substances using a so-called filter trap assay, in which the samples of the oxidized enzyme in solution containing sodium dodecyl sulfate with the addition of chemicals are subjected to ultrafiltration in a 96-well manifold. In the screening, five compounds were selected, two of which were shown to prevent oxidative stress-induced cell death (Table 2). A couple of compounds were tested in a malonate-induced model of oxidative stress in rats and demonstrated the ability to reduce the region of neuronal loss in the brain, according to MRI data, and to compensate the defects in motility of rats [118]. Further studies showed that one of the above compounds, a hydrocortisone derivative (RX624), was able to inhibit the intercellular propagation of polyglutamine pathology [3] and the expansion of neuronal loss in the brains of animals after physical trauma [28].

In regards to pathologies stemming from proteins capable of forming toxic oligomers or aggregates, two approaches for the search of compounds targeting GAPDH may be employed. Firstly, the substances can be used that are able to penetrate cells and prevent the formation of pathogenic protein complexes containing GAPDH. Such aggregates may include mutant proteins or polypeptides damaged by common toxic factors like, for example, oxidative stress. When choosing such compounds, it is necessary to opt for those which do not affect the glycolytic function of GAPDH and do not have high toxicity. Peptides, for example, GAI [117], may be the most promising in this implication. Secondly, there are compounds with high affinity to GAPDH, but for which the ability to penetrate the cell is not approved. As a rule, such substances possess low toxicity and may be effective in combating the effects caused by massive death of brain cells [3]. The main mechanism of action of such compounds is the suppression of the cytotoxicity exerted by exogenous protein complexes, as was shown for RX-624 able to affect both aggregating GAPDH or that in complex with polyglutamine [119]. Further studies are needed to demonstrate the advantages of the above approaches to suppress the pathogenic effects of modified and aggregating GAPDH.

It is believed that drugs inhibiting the pro-apoptotic effects of GAPDH should reduce its ability for nuclear translocation and/or prevent its interactions with other molecules, proteins or nucleic acids. Since GAPDH is suggested to be a key transcriptional coactivator necessary for entry into the S phase, Xing and coauthors assumed that reducing GAPDH’s binding to DNA may be toxic. They used saframycin A, known to form a complex with duplex DNA, and found that GAPDH possessed DNA-binding activity and formed a ternary complex with saframycin A and DNA that induces a toxic response in tumor cells [120], see Table 2. Another potential anti-tumor drug targeting GAPDH is a natural chemical, koningic acid, which was selected using an advanced approach based on the analysis of the rate-controlling enzymes during the Warburg effect. This approach allowed predicting a response to factors targeting glucose metabolism and the application of molecular docking proved koningic acid to be a selective inhibitor of GAPDH [99]. One more compound aimed at blocking the glycolytic activity of GAPDH is DC-5163. The use of this drug inhibited proliferation and induced apoptosis in MDA-MB-231 cells [121].

The impact of GAPDH on the efficacy of metabolism in cancer cells may be also regulated by the coactivator-associated arginine methyltransferase 1 (CARM1 or PRMT4), an enzyme methylating arginine-234 on the GAPDH molecule. This amino acid is located inside the catalytic site of GAPDH, and its methylation leads to the inhibition of GAPDH activity and to the reduction of liver cancer tumorigenicity in vitro and in vivo [126]. Fairly similar data were obtained when analyzing CARM1-mediated methylation of another enzyme, malate dehydrogenase. Methylation of arginine 248 led to the suppression of mitochondria respiration and the inhibition of glutamine metabolism, which sensitized pancreatic ductal adenocarcinoma cells to oxidative stress and strongly decreased cell proliferation [127]. Controversially, methylation of the key glycolytic enzyme pyruvate kinase M2 isoform (PKM2) by CARM1 was found to switch the balance of metabolism from oxidative phosphorylation to aerobic glycolysis in breast cancer cells. In this experimental scheme, inhibition of PKM2 methylation using competitive peptide caused the reduction of breast cancer cell proliferation, migration and metastasis [46].

Another compound which affects the GAPDH function in glycolysis is a derivative of the Krebs cycle intermediate fumarate, dimethyl fumarate, a serendipitously discovered immunomodulatory drug used to treat psoriasis and multiple sclerosis (see Table 2). This compound succinated C152 on GAPDH molecule, inactivated catalytic cysteine of GAPDH in mice and in humans, both in vitro and in vivo, reduced aerobic glycolysis in activated—but not resting—myeloid and lymphoid cells and exhibited an anti-inflammatory effect for mouse peritoneal macrophages. The authors suggest that fumarate-induced inactivation of GAPDH by dimethyl fumarate, which is a more cell-permeable and electrophilic derivative of fumarate, may explain its utility in the treatment of a variety of well-spread autoimmune pathologies [40].

Among the compounds able to inhibit multiple functions of GAPDH, the triazine-based substance GAPDS is worth mentioning. It possesses anti-cancer activity which is associated with the inhibition of the GAPDH-mediated effect on microtubule formation (Table 2). The authors showed that GAPDS treatment caused inhibition of actin filament and microtubule formation in vitro and loss of motility of tumor cells belonging to different lineages [125]. In the HCT-116 colon carcinoma xenograft model based on zebra fish, GAPDS demonstrated a strong reduction of tumor dissemination.

In the aspect of oncological diseases, it is obvious that there is an acute issue of targeted delivery of GAPDH glycolytic activity inhibitors, for example, using liposomes. Nevertheless, we understand that such studies have not yet been carried out, as well as the GAPDH-targeted antitumor compounds not have been used in the clinic so far.

## 4. Conclusions

In conclusion, the multiplicity of functions of GAPDH in pathologies, from cancer to neurodegenerative and autoimmune diseases, demands appropriate therapeutic methods of the modulation of the enzyme function. This means that the search for GAPDH-targeted medicines should be performed in accordance with the certain deviation of the enzyme function in a given pathology and in the connection with structural features of its molecule. We suggest that some of the GAPDH-targeted medicines could be an integral part of the combination therapy of socially significant diseases.

## Figures and Tables

**Table 1:** Glyceraldehyde-3-phosphate dehydrogenase (GAPDH) as an important participant of pathological processes.

	Pathology	Mechanism	Reference
Pathologies associated with pro-apoptotic GAPDH functioning	Secondary damage after traumatic brain injury	Formation of exogenous cytotoxic aggregates	[28]
	Parkinson’s disease	α-synuclein binding	[29]
	Alzheimer’s disease	Participation in formation of cytotoxic aggregates	[30]
	Huntington’s disease	Participation in formation of cytotoxic aggregates	[31,32]
	Diabetic retinopathy	Increased GAPDH nuclear translocation in retinal cells	[33]
	Amyotrophic lateral sclerosis	Participation in formation of cytotoxic aggregates	[34]
Pathologies associated with impaired function of GAPDH	Senescence	Inhibition of telomerase activity and shortening of telomere length	[35]
	DRPLA	Glycolysis impairment	[36]
	Male infertility	Immune response to sperm-specific GAPDH	[37,38]
	Hepatocellular carcinoma	Involvement of GAPDH in viral hepatitis, metabolic alteration	[39]
	Autoimmune diseases	Post-transcriptional regulation of cytokine production	[40]
	Age-related nuclear cataract	Reducing of GAPDH expression	[41]
	Secondary damage after ischemic renal injury	Glycolysis impairment	[42]
	Huntington’s disease	Violation of GAPDH-mediated mitophagy	[43]
	Septic shock	Upregulation of TNF translation	[44]
Pro-survival function of GAPDH in cancer cells	Non-Hodgkin’s B lymphomas	Promotion of NF-κB-dependent induction of HIF-1α	[45]
	Colon cancer metastasis	Upregulation of Snail expression	[46]
	Ovarian cancer	Inhibition of apoptosis	[47]

**Table 2:** GAPDH-targeted therapeutic agents.

Name	Pathology, Clinical Verification (Approval)	Mechanism of Action	Reference
(*S*)-1-[*N*-(4-chlorobenzyl) succinamoyl] pyrrolidine-2-carbaldehyde (ONO-1603)	Age-related dementia	Endopeptidase inhibitor, Inhibitor of GAPDH gene expression	[110]
Decapeptide GAI	Neurodegenerative pathologies	Reduce GAPDH aggregation	[117]
Deprenyl	Huntington’s disease	Block GAPDH recruitment into polyQ aggregates	[32]
Dimethyl fumarate	Autoimmne diseases	Covalently modifies cysteine residues	[40]
Iodoacetate	Colon carcinoma	Inhibitor of GAPDH glycolytic activity	[122]
Koningic acid	Cancers	Inhibitor of GAPDH (molecular docking)	[99]
DC-5163	Epithelial tumors	Inhibitor of GAPDH glycolytic activity	[121]
Methylglyoxal	Diabetes	Increasing of GAPDH glycation	[123]
Hydrocortisone 21-hemisuccinate (RX624)	Huntington’s disease, traumatic brain injury	Reduce GAPDH aggregation	[3,28]
Saframycin A	Cancer	Induce GAPDH nuclear translocation	[120]
TAT-GluA2NT1-3-2 peptide	Epilepsy	Inhibition of exitotoxicity	[124]
Tetrahydroaminoacridine (tacrine)	Alzheimer’s disease	Inhibitor of GAPDH gene expression	[109]
Triazine, GAPDS	Colon carcinoma, cervical carcinoma	Reduce GAPDH levels in the cytoplasm	[125]
